# Extended cleavage specificities of human granzymes A and K, two closely related enzymes with conserved but still poorly defined functions in T and NK cell-mediated immunity

**DOI:** 10.3389/fimmu.2023.1211295

**Published:** 2023-07-11

**Authors:** Erdem Aybay, Jinhye Ryu, Zhirong Fu, Srinivas Akula, Erika Mendez Enriquez, Jenny Hallgren, Sara Wernersson, Anna-Karin Olsson, Lars Hellman

**Affiliations:** ^1^ Department of Cell and Molecular Biology, Uppsala University, Uppsala, The Biomedical Center, Uppsala, Sweden; ^2^ Department of Anatomy, Physiology, and Biochemistry, Swedish University of Agricultural Sciences, Uppsala, Sweden; ^3^ Department of Medical Biochemistry and Microbiology, The Biomedical Center, Uppsala, Sweden

**Keywords:** cytotoxic T Cells, NK cell, granzyme, apoptosis, caspase, cytokines

## Abstract

Granzymes A and K are two highly homologous serine proteases expressed by mammalian cytotoxic T cells (CTL) and natural killer cells (NK). Granzyme A is the most abundant of the different granzymes (gzms) expressed by these two cell types. Gzms A and K are found in all jawed vertebrates and are the most well conserved of all hematopoietic serine proteases. Their potential functions have been studied extensively for many years, however, without clear conclusions. Gzm A was for many years thought to serve as a key component in the defense against viral infection by the induction of apoptosis in virus-infected cells, similar to gzm B. However, later studies have questioned this role and instead indicated that gzm A may act as a potent inducer of inflammatory cytokines and chemokines. Gzms A and K form clearly separate branches in a phylogenetic tree indicating separate functions. Transcriptional analyses presented here demonstrate the presence of gzm A and K transcripts in both CD4^+^ and CD8^+^ T cells. To enable screening for their primary biological targets we have made a detailed analysis of their extended cleavage specificities. Phage display analysis of the cleavage specificity of the recombinant enzymes showed that both gzms A and K are strict tryptases with high selectivity for Arg over Lys in the P1 position. The major differences in the specificities of these two enzymes are located N-terminally of the cleavage site, where gzm A prefers small amino acids such as Gly in the P3 position and shows a relatively relaxed selectivity in the P2 position. In contrast, gzm K prefers large amino acids such as Phe, Tyr, and Trp in both the P2 and P3 positions and does not tolerate negatively charged residues in the P2 position. This major distinction in extended specificities is likely reflected also in preferred *in vivo* targets of these two enzymes. This information can now be utilized for high-precision screening of primary targets for gzms A and K in search of their highly conserved but still poorly defined functions in vertebrate immunity.

## 1 Introduction

Cytotoxic T cells (CTLs) and natural killer cells (NK cells) play very important roles in the protection against intracellular pathogens. CTLs recognize infected cells by MHC-T-cell receptor (TCR) interaction whereas NK-cells recognize these cells by their lack of MHC class I, or by antibody-dependent cellular cytotoxicity (ADCC). Following the direct cell-to-cell contact, CTLs and NK cells induce an apoptosis signal in the target cell by FAS-FAS-ligand interaction and by delivering serine proteases into the target cell that can activate the cellular apoptosis machinery. The delivery of these serine proteases is dependent on the pore-forming molecule perforin, that is homologous to one of the complement components, the C9 (1, 2). Perforin and the abundant serine proteases are stored in cytoplasmic granules for rapid release upon contact with cells infected with intracellular pathogens. These serine proteases, which are stored in their active form within the granules, belong to the large family of chymotrypsin-related serine proteases (3, 4). The serine proteases expressed by CTLs and NK cells are named granzymes (gzms), due to their granule storage. The best characterized of them is gzm B, which is involved in both caspase-dependent and independent activation of apoptosis in virus-infected cells (5–7). The gene coding for gzm B (or a gzm B-like protease) is positioned in the chymase locus and is found in all tetrapods, from amphibians to humans (8–11). Gzm B, has Asp-ase activity, and the human chymase locus also includes gzm H, which has chymotryptic cleavage specificity (8, 12). Three other gzms are also found in human CTLs and NK cells, the gzms M, A, and K. Gzm M is located in the met-ase locus together with several of the major neutrophil proteases, whereas gzms A and K are located in the T cell tryptase locus, also named the gzm A/K locus (4).

Gzm A is the most highly expressed of the different gzms and has been studied extensively for many years. Initial studies indicated a role in apoptosis induction similar to what has been shown for gzm B (13). However, later studies using more physiological, nanomolar, concentrations of gzm A indicated that this enzyme is not involved in the induction of apoptosis but instead induce the expression of several inflammatory cytokines and chemokines (7). Interestingly, this cytokine induction was primarily observed in monocytes and macrophages and included primarily the classical inflammatory cytokines and chemokines, IL-1, IL-6, TNF-α and IL-8 (7). Contradicting information concerning the role of the proteolytic activity of gzm A in this induction has also come from different labs. In the initial study, Metkar et al. showed that the inactive enzyme did not induce cytokine/chemokine expression, whereas in later studies Wensik et al. showed that also an inactive enzyme could have this effect (7, 14). The mechanisms by which gzm A could induce this cytokine and chemokine release and the potential difference in function between gzms A and K are also still not resolved. Thereby, a number of questions still remain unanswered concerning these highly conserved enzymes.

Both gzm A and K seem to have effects both extracellularly and intracellularly, and elevated levels of these enzymes have been observed upon infection with several types of pathogens (bacteria, viruses, and parasites), indicating a complex role in immunity (15–19). One way to obtain a better understanding of the function of these enzymes is to study their extended cleavage specificity. Analysis of the structure of a large number of hematopoietic serine proteases has shown that amino acids 189, 216 and 226 (according to bovine chymotrypsin numbering), which line the active site pocket, are responsible for the primary specificity of the enzyme (20). Both gzms A and K have the triplet DGG (aspartic acid, glycine, and glycine). Such a triplet indicates that these enzymes have trypsin-like activity, with a preference for basic amino acids, such as arginine and lysine, in their P1 position (the amino acid after which the enzyme cleaves). Studies using both peptide libraries and proteomic analysis have previously resulted in a relatively detailed view of their cleavage specificities (21–23). However, there are still major questions concerning the differences in specificity between gzms A and K, and their primary *in vivo* targets remain unknown (21–23). To obtain a better picture of potential differences in the target selection by gzms A and K we therefore performed a detailed analysis of their extended cleavage specificities by phage display and a panel of recombinant substrates. Our analysis shows that these two proteases, despite their very high primary sequence similarity, differ markedly in their extended specificities. Both have the same primary specificity, with a high selectivity for arginine in the P1 position of a substrate, but they differ markedly in the selectivity of preferred amino acids in positions N-terminally of the cleavage site. This clearly indicates different *in vivo* targets for these two enzymes. This detailed information concerning their target selectivity can now be used in the screening for the most important *in vivo* targets. Identification of such targets can potentially resolve the many remaining questions concerning the primary biological functions of gzms A and K.

## 2 Materials and methods

### 2.1 Production and purification of recombinant human gzms A and K

The sequences of human gzms A and K were retrieved from the NCBI database with the following accession numbers, human GZMA (NM_006144), human GZMK (NM_002104). The expression constructs for these two proteases were ordered as designer genes from GenScript (Piscataway, NJ, USA). The construct contains a signal sequence, an N-terminal His_6_-tag, and an enterokinase (EK) site (Signal sequence-HHHHHHDDDDK-active protease). The His-tag is used for purification purposes and the EK site is for activation of the enzyme by removing the charged N-terminal region including the His_6_-tag and the EK site by EK cleavage. These sequences were after synthesis inserted in the mammalian expression vector pCEP-Pu2 by Genscript and sequence verified (24). The vectors were transfected into the human embryonic kidney cell line HEK293-EBNA for expression of the recombinant enzyme. After purification on IMAC Ni ^2+^ agarose, the enzymes were activated by the addition of 1µl enterokinase into 90µl of the eluted recombinant protein (Roche, Mannheim, Germany). The sample was mixed, followed by a 37°C incubation for 5 hours to activate the protease. The purity and activation of these two enzymes were then determined by separation on 4-12% SDS-PAGE gels (Invitrogen, Carlsbad, CA, USA). 2.5µl Sample Buffer, containing Sodium dodecyl sulfate (SDS), and 0.5µl β-mercapto-ethanol were added to 10µl protein followed by 85°C heating and SDS-PAGE gel electrophoresis. Overnight staining in colloidal Coomassie staining solution followed by de-staining by several washes enable the visualization of the protein bands (25).

### 2.2 Determination of cleavage specificity by phage-displayed nonapeptide library

A library of T7 phages was designed to contain a random nonameric peptide followed by a His_6_-tag at the C-terminus of the capsid protein. This library, containing approximately 50 million independent phages, was used to determine the cleavage specificity of the human gzms A and K. 250 µl nickel–nitriloacetic acid (Ni–NTA) beads were used to immobilize the phages based on the interaction with His_6_-tags by mixing and gentle agitation at 4°C for 1 hour. 1.5 ml of washing buffer (1M NaCl, 0.1% Tween-20 in PBS, pH 7.2) was then added to remove unbound phages. This was repeated 10 times to ensure proper washing. The beads were then washed two times in 1.5 ml PBS and suspended in PBS in a total volume of 500 µl. An appropriate amount of recombinant protease was then added into the Eppendorf tubes followed by gentle agitation at 37°C for approximately 16 hours (Over-night). One tube without protease was used as a control. Phages containing peptide sequences which are cleavable by the protease were released from Ni–NTA beads and these phages can be separated from the still uncleaved and thereby bead-bound phages by centrifugation, enabling the recovery of the cleaved phages from the supernatant. 15 µl Ni–NTA beads were added to the supernatant in order to remove phages that still had His_6_-tags but had been released by other means than cleavage ensuring that the His_6_-tags had been removed on all the phages in the supernatant. 100 µl of 100 mM imidazole was used to elute the phages from the remaining Ni–NTA beads to determine the number of phages initially bound. In order to determine the number the phages detached from the Ni–NTA beads by the protease, a series of dilutions of the supernatant were plated onto LA-Amp plates together with 3 ml of 0.6% top agarose, 300 µl of *Escherichia coli* (BLT 5615) and 100 µl of 100 mM isopropyl β-D-1-thiogalactopyranoside (IPTG). Ten ml of Escherichia coli (BLT 5615) was prepared to amplify the remaining phages for the next round of selection. To this culture 100 µl, of a 100 mM solution of IPTG was added to induce the expression of T7 phage capsid protein. The bacteria were lysed after approximately 2 hours of gentle agitation at 37°C, and the lysate was centrifuged to remove cell debris before entering the next round of selection. After 5-7 rounds of selection, 110 plaques were picked by glass Pasteur pipettes from LA-Amp plates after plating in top agarose and the plugs were transferred into Eppendorf tubes containing phage extraction buffer (100 mM NaCl and 6 mM MgSO_4_ in 20 mM Tris-HCl, pH 8.0). Vigorous shaking for 30 minutes at 4°C was critical for extracting the phages from the agarose. Polymerase chain reaction (PCR) was then used to amplify an approximately 300 bp region of phage DNA encoding the random nonamer region. The quality and quantity of the amplified DNA were determined by gel electrophoresis and the 96 samples with the best DNA quality were sent in a microtiter plate for sequencing to Eurofins (Ebersberg, Germany).

### 2.3 Generation of a consensus sequence from sequenced phage inserts

The results from the sequence analysis were translated into amino acid sequences by CLC Sequence viewer 8. The amino acid sequences were then aligned by hand, looking for sequence patterns, based on the results of previous studies of similar enzymes and from analysis of primary specificity by cleavage of chromogenic substrates, which has shown high selectivity for arginine in the P1 position. Amino acids were classified based on the particular characteristics as follows: aromatic amino acid (Phe, Tyr, Trp); negatively charged amino acid (Asp, Glu); positively charged amino acid (Lys, Arg); small aliphatic amino acid (Gly, Ala); larger aliphatic amino acid (Val, Leu, Ile, Pro) and other amino acids (Ser, Thr, His, Asn, Gln, Cys, Met). The peptide bond between P1 and P1΄position represents the scissile bond, based on the nomenclature by Schechter and Berger to designate the substrate cleavage region (20).

The sequences from the phage display were also added to a file in the Web-logo program to generate a graphical presentation of the selectivity of these two enzymes (https://weblogo.berkeley.edu/logo.cgi).

### 2.4 Generation of recombinant substrates for the analysis of the cleavage specificity

To verify the result from the phage display and obtain quantitative information concerning the importance of particular amino acids at positions at and around the cleavage site we use recombinant substrates. The vector pET21 has been modified to carry two copies of the E. coli thioredoxin (Trx) gene for bacterial expression. A His_6_-tag has been inserted in the C-terminal end of the second copy of the Trx for purification with Ni–NTA beads. Substrate sequences are then inserted between the two thioredoxin molecules by the use of two complementary oligonucleotides. The oligonucleotides were ordered to match the sticky ends of two unique restriction sites (BamHI and SalI) followed by ligation and cloning. Individual clones were picked and sequenced to ensure the correct linker sequence between the two Trx molecules inserted by the oligos. The verified plasmids were then transformed into the *E. coli Rosetta Gami* strain for protein expression (Novagen, Merck, Darmstadt, Germany). The transformed strains were grown overnight. In the morning 10 ml of the culture was added to 90 ml of LB-Amp in a new E-flask and incubated at 37°C for 1 hour until the OD (600 nm) reached 0.5. The culture was then induced by the addition of 1 ml of a 100 mM solution of IPTG to a final concentration of 1 mM and incubated at 37°C for an additional 3 hours under vigorous shaking. The bacteria were centrifuged at 3000 rpm for 12 minutes at 4°C, resuspended in 25 ml PBS containing 0.05% Tween followed by an additional centrifugation where-after the pellet was dissolved in 2 ml PBS. Sonication was then used to open the cells to release their cellular contents. Cooling on ice after every 30 seconds of sonication was important for avoiding overheating. Sonication 5 times for 30 seconds each time was performed to ensure efficient breaking of the cell wall. The debris was removed by centrifugation at 13,000 rpm for 3 minutes at 4°C and the supernatant was transferred into new tubes. 250 µl Ni–NTA slurry (50:50; Qiagen, Hilden, Germany) was added and the samples were incubated for 45 minutes in cold room under slow rotation. The Ni–NTA beads were then transferred into a 2 ml syringe with a glass filter in the bottom of the syringe. The beads were washed three times with 1 ml, and two times with 2 ml of washing buffer (PBS, 0.05% Tween 20, 10 mM Imidazole, and 1 M NaCl), and the protein was then eluted in six fractions 100 µl (for the first fraction) and 200 µl (for the following fractions) of elution buffer (PBS, 0.05% Tween 20, 100 mM imidazole). Each eluted fraction was analyzed on SDS Bis-Tris 4-12% PAGE gel by mixing 10 µl of protein sample with 2.5 µl of a 4x sample buffer and 0.5µl β-mercapto-ethanol followed by heating for 8 minutes at 85°C. The protein concentration for each fraction was estimated by comparing with a control sample for which the concentration was already known. The fractions were diluted into similar concentrations as the control sample and 10 µl of the dilutions were mixed with 1 µl diluted gzm A or K (based on the activity of the enzyme) leaving at room temperature for the reaction at time points 0, 15, 45 and 150 minutes, respectively. Sample buffer was used to stop the reaction and 0.5 µl of β-mercapto-ethanol was then added to each sample before heating for 8 minutes at 85°C. The results from the cleavage reactions were analyzed on 4-12% SDS-PAGE gels (Invitrogen, Carlsbad, CA, USA) followed by staining overnight in colloidal Coomassie staining solution and de-stained, first in 30% methanol/water solution and then in water for several times (25).

### 2.5 Analysis of the cytokine inducing activity of recombinant gzm A and the possible presence of LPS in the gzm A preparation

To determine the potential cytokine inducing activity of active human gzm A on purified human peripheral blood monocytes we followed the protocol presented in a recently published article on the effect of LPS on human monocytes where both the cell isolation, culture conditions and the transcriptome analysis is described in detail (26). Ten ug of active recombinant human gzm A in 100ul PBS was added to the gzm A cultures. These cultures were then incubated at 37°C for 4 hours before harvesting and analysis by ThermoFisher Ampliseq technology of their transcriptome.

To determine the amount of potential contaminating LPS in the recombinant gzm A preparation we used the Pierce commercial Chromogenic Endotoxin Quant kit A39552S according to the manufacturer’s recommendation (ThermoFisher, Waltham, MA USA).

### 2.6 Isolation of human peripheral blood B cells, monocytes, and CD4^+^ and CD8^+^ T cells by FACS sorting

Blood samples collected from buffy coats from anonymized blood donors at Uppsala University hospital were used to enrich peripheral blood mononuclear cells (PBMC). PBMC were enriched using Ficoll-Paque Premium (ρ=1.076 g/ml) (GE Healthcare, Little Chalfont, UK) in SepMate™-50 tubes (Stemcell Technologies, Vancouver, Canada). Platelets were removed by centrifugation (2 x 200g, 10 minutes). Enriched PBMC were frozen and kept at -80 °C in heat inactivated fetal calf serum (Sigma-Aldrich, St. Louis, MO, USA) with 10% of DMSO. On the day of the cell sorting the enriched cryopreserved PBMCs were after thawing incubated in PBS, pH 7.4 with 2% heat inactivated fetal calf serum containing the following fluorescent-conjugated antibodies targeting CD4 (RPA-T4), CD8 (RPA-T8), CD14 (M5E2), CD19 (HIB19) from BD Bioscience and eBioscience, San Diego, CA, USA. For the Ampliseq analysis, the cells (10^6^ cells of each population) were collected in PBS, pH 7.4 with 2% heat inactivated fetal calf serum and kept on ice until RNA extraction. The cell sorting was performed on a FACSAria III (BD Biosciences). Data analysis was performed using FlowJo software version 9.8.

### 2.7 Ampliseq analysis of the total transcriptome

The isolated cells were pelleted and total RNA was prepared from the cell pellets using the RNeasy Plus mini kit (Qiagen, Hilden, Germany), according to the manufacturer´s recommendations. The RNA was eluted with 30 μl of DEPC-treated water, and the concentration of RNA was determined by using a Nanodrop ND-1000 (Nano Drop Technologies, Wilmington, Delaware, USA). The integrity of the RNA was confirmed by visualization on 1.2% agarose gel using ethidium bromide staining.

The freshly isolated RNA was analyzed for their total transcriptome by the Thermo-Fisher chip based Ampliseq transcriptomic platform at the SciLife lab in Uppsala, Sweden (Ion-Torrent next-generation sequencing system- Thermofisher.com). The sequence results were delivered in the form of a Excel file.

## 3 Results

### 3.1 Phylogenetic analyses of the gzm A/K locus

**Figure 1:**
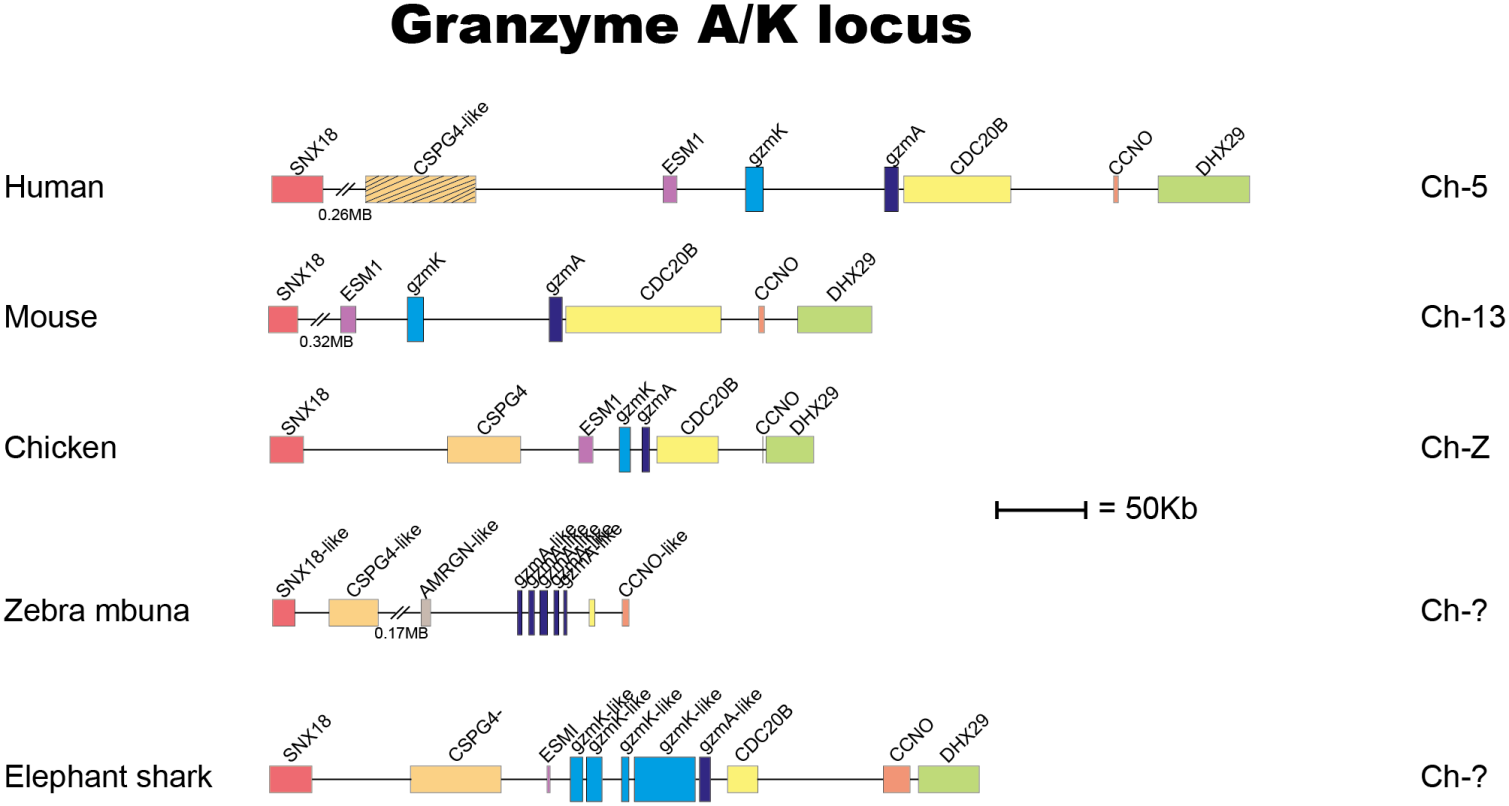
The gene locus encoding gzms A and K. In almost all jawed vertebrates the gzms A and K are encoded from the gzm A/K locus also named the T cell tryptase locus. Gzm A related genes are marked in dark blue, and the gzm K related gene is in light blue. The protease genes are in double hight compared to the surrounding non-protease genes for an easier identification.

**Figure 2:**
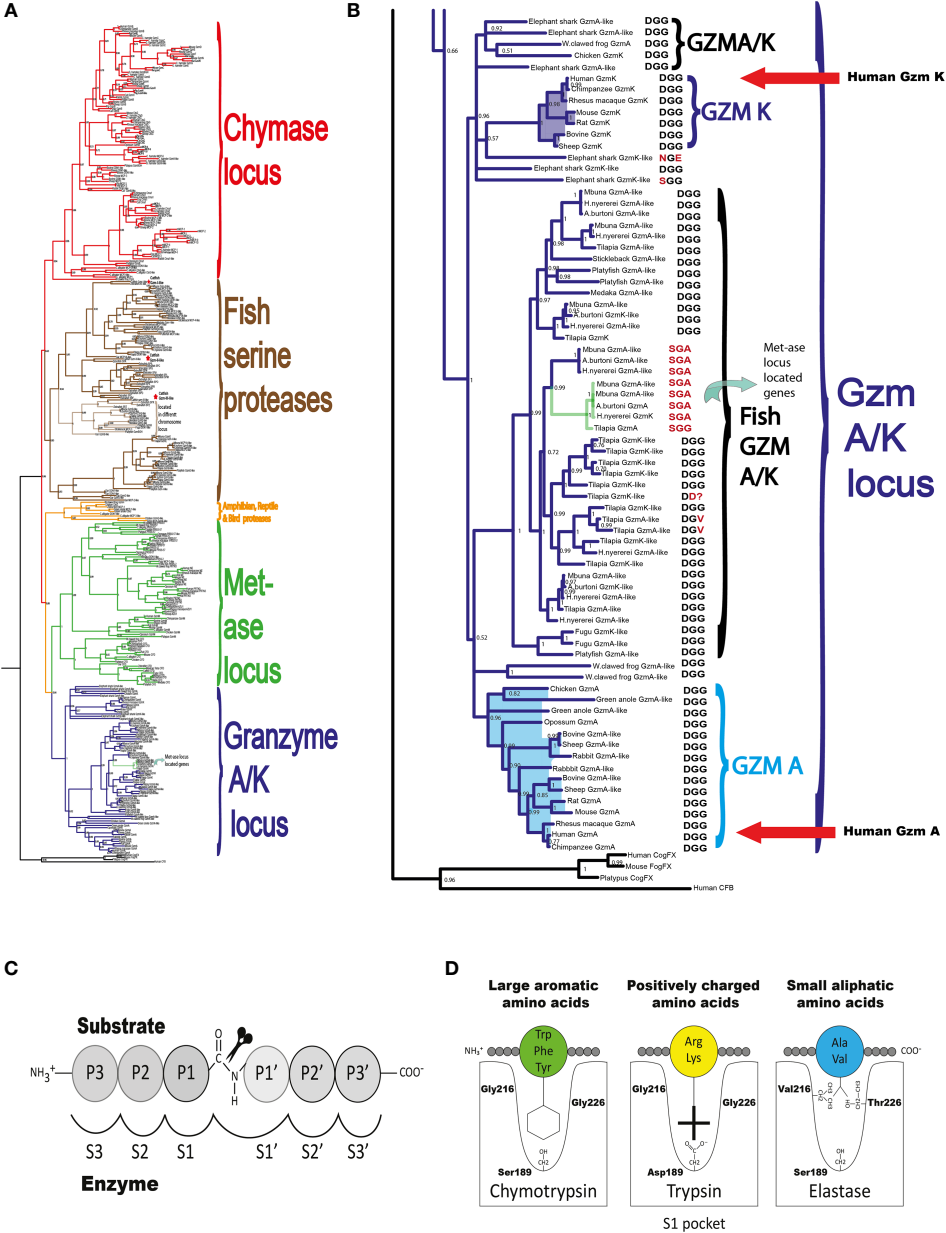
A phylogenetic tree of gzm A/K locus genes. A phylogenetic tree was constructed based on the relationships among gzm A/K locus genes of a number of mammals using both MrBase analysis program and Maximum-likelihood algorithm using the manual standard protocol (27). The phylogenetic trees were drawn in FigTree 1.4.2 (http://tree.bio.ed.ac.uk/software/figtre​e/). In **(A)** we show the phylogenetic tree of a large number of hematopoietic serine protease genes originating from the five major loci. As can be seen from the figure the genes from the different loci cluster together. One branch for chymase locus encoded gene, marked in red, one branch for met-ase locus genes, marked in green, one branch for the major fish serine proteases marked in brown, a separate small branch for a locus found in amphibians, reptiles, and birds but not in mammals marked in orange and finally a branch marked in dark purple with all the gzm A/K genes. In **(B)** we show an enlarged picture of the genes of the gzm A/K locus with the enzymes of major interest for this study and that have been analyzed by phage display are marked by red arrows. In **(C, D)** we show the nomenclature of the amino acids surrounding the cleaved peptide bond and the amino acids forming the active site pocket. **(C)** shows the amino acids N-terminal from the cleaved bond are termed P1 (where cleavage occurs, depicted by scissors), P2, P3, etc. Amino acids C-terminal of the cleaved bond are termed P1’ (adjacent to P1), P2’, P3’ etc. The corresponding interacting sub-sites in the enzyme are denoted with S. **(D)** shows the S1 pocket, which is important in determining the primary specificity of the chymotrypsin family. The important residues are shown, which determine chymotrypsin-, trypsin- or elastase-like specificity. Three residues corresponding to positions 189, 216, and 226 in bovine pancreatic chymotrypsinogen have been found to be the amino acids forming the major part of this pocket and thereby giving the primary specificity of the enzyme (20). These three amino acids are marked, in one letter code, after each protease in **(B)** Almost all of these enzymes have the DGG triplet, indicating tryptase specificity.

The locus encoding gzm A/K is the first of these loci to appear during vertebrate evolution (4). This locus is found in all jawed vertebrates analyzed, from cartilaginous fishes to humans with a relatively similar organization (**Figure 1**) (4). By analyzing a large panel of different vertebrate hematopoietic serine proteases, we can see that the genes encoded from the gzm A/K locus form a separate subfamily in a phylogenetic tree (**Figures 2A, B**). These studies also revealed that the absolute majority of these enzymes have the triplet DGG (aspartic acid, glycine, and glycine). Such a triplet indicates that these enzymes have trypsin-like activity, with a preference for basic amino acids, such as arginine and lysine, in their P1 position (the amino acid after which the enzyme cleaves) (**Figure 2C**). This is primarily due to the presence of the negatively charged aspartic acid in position 189 at the bottom of the cleft (**Figure 2D**), which favors positively charged amino acids in the substrate by electrostatic interaction. The majority of all gzm A and Ks are, based on the amino acids of their active site, tryptases (**Figure 2B**). However, there is a small family of gzm A/K members that have another triplet, SGA, which indicates that they display chymotrypsin-like activity (**Figure 2B**). All of these genes are found in a few cichlid species indicating that this change in primary specificity occurred in an early member of the A/K locus in this fish lineage. Among the other members, which all seem to be tryptases, the mammalian gzm Ks form a separate sub-branch on the gzm A/K tree, indicating that they form a functional subfamily. Thus, we hypothesized that analysis of extended cleavage specificity of both human gzm A and K could determine whether such functional diversification has occurred.

### 3.2 Purification and activation of recombinant human gzms A and K

**Figure 3:**
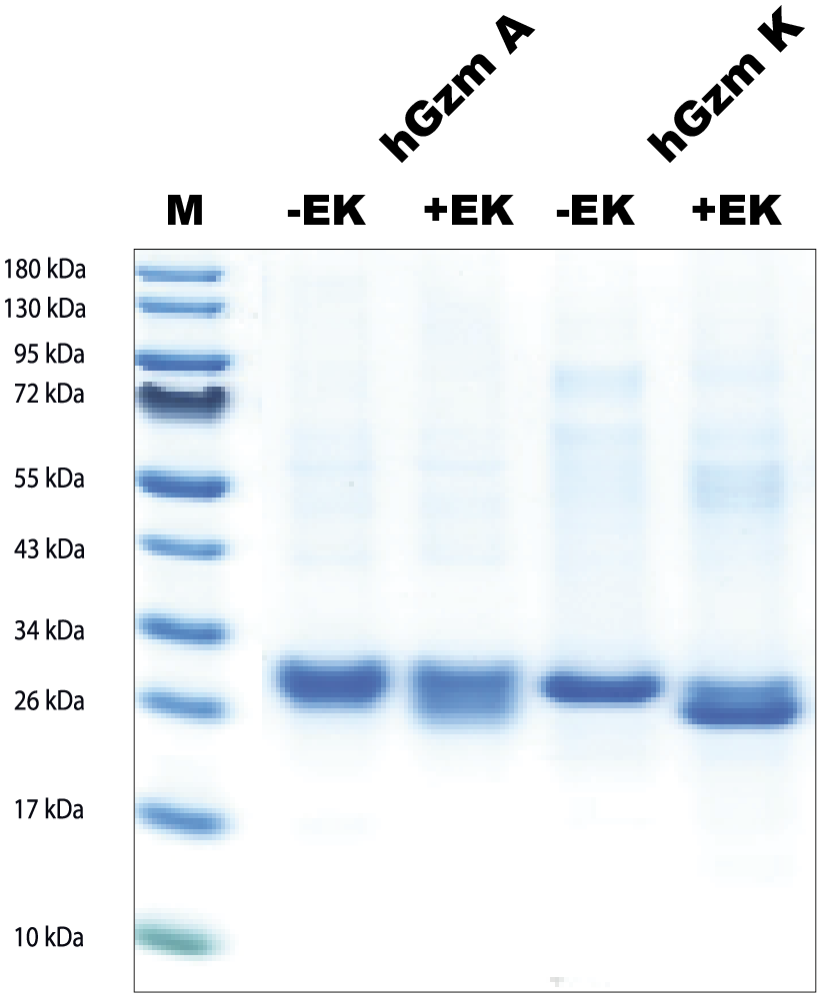
SDS-PAGE gel separation of human gzms A and K used in this study. The human gzms A and K were produced as inactive enzymes containing an N terminal His_6_-tag and an enterokinase site. The enzymes were both produced in the human cell line HEK293-EBNA with the episomal vector pCEP-Pu2. Entrokinase (EK) was used to cleave of the N-terminal tail to activate the two enzymes. The inactive enzymes with the N-terminal purification tag and the active enzymes were analyzed by separation on SDS-PAGE gel and visualized with Coomassie Brilliant Blue staining. PAGE Ruler was used as marker. M, marker; +EK, with entero-kinase; -EK, without enterokinase.

The sequences for the coding regions of human gzms A and K were obtained from the NCBI database and the sequences were ordered as designer genes. These designer genes were inserted into the pCEP-Pu2 vector and sequence verified (24). In front of the first codon of the active granzyme, we inserted the coding region for six histidines (a His_6_-tag) and an enterokinase site (Asp-Asp-Asp-Asp-Lys). Following purification of the enzymes from the conditioned media on Ni-NTA agarose the enzymes were activated by the cleavage with enterokinase to remove the His_6_-tag and the enterokinase site. The cleavage results in the reduction in size of the protein by 1.5-2kD, which was confirmed by SDS-PAGE analysis (**Figure 3**).

### 3.3 Determination of the extended cleavage specificity by substrate phage display

**Figure 4:**
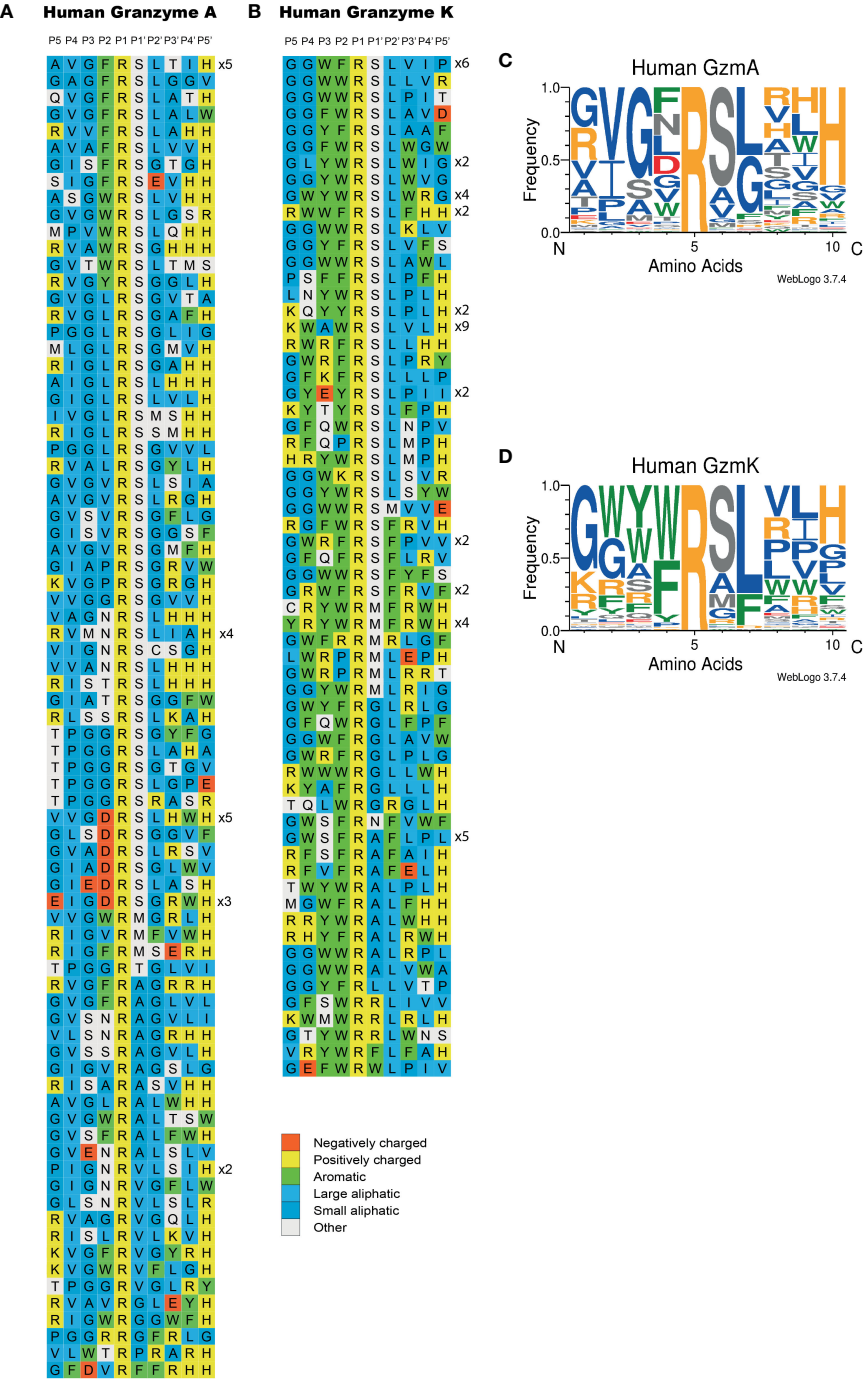
Phage display sequences generated after cleavage by human gzms A and K after six rounds of selection. After the last round of selection, the released phages were collected for sequencing. The amino acid sequences are P-G-G-?-?-?-?-?-?-?-?-?-H-H-H-H-H-H and the “?” means randomized amino acids. The amino acid sequences were aligned into a P5-P4’ consensus. The cleavage occurs between positions P1 and P1’. The classification of amino acids was indicated by color based on the side chain properties located in the right bottom corner of the figure. In **(A)** we show the results for gzm A and in **(B)** for gzm K Web Logo presentations of the target selectivity of human gzms A and K are displayed in **(C, D)**, respectively.

A library of T7 phages was used to determine the extended cleavage specificities of human gzms A and K. The phage library used contains approximately 50 million phage clones and each clone expresses a unique random sequence of 9 amino acids. Analysis of the cleavage specificity by these two enzymes were performed over 6 rounds of pannings and the result showed approximately 77 and 140 times enrichment of phages compared to the PBS negative control for gzms A and K, respectively. One hundred and ten individual phage colonies were picked for each of the two proteases and a region of approximately 300bp including the coding region for the 9 amino acid random region was amplified by PCR. Ninety-six of the PCR products with the most distinct PCR bands for gzm A and ninety-six for K were sent for sequencing. The nucleotide sequence was translated into amino acid sequence and the 9 random amino acid region of each sequence was aligned by hand based on common sequence characteristics (**Figure 4**).

Both human gzms A and K were found to show a strict preference for Arg over Lys in the P1 position, as all of the clones had an Arg in the P1 position (**Figure 4**). For both enzymes, a relatively strong preference for Ser was seen in the P1´position (65% and 52%, **Figure 4**). The P1´position is the amino acid just C-terminally of the cleaved bond (**Figure 2C**). A very strong preference for Leu in the P2´position was observed for gzm K (78%) and a weaker selectivity of aliphatic amino acids in this position was seen for gzm A (**Figure 4**). In positions P3´ and P4´, there was a selection for aliphatic and aromatic amino acids, but also for positively charged amino acids for both enzymes (**Figure 4**). The major differences between the two enzymes, except for the preference for Leu in the P2´ position for gzm K, were seen in positions N-terminally of the cleavage site. Gzm K showed a very strong preference for aromatic amino acids particularly in the P2 and P3 positions but also in the P4 position (92%, 65% and 44%, respectively, **Figure 4**). In contrast, gzm A showed a strong preference for the small amino acid Gly in the P3 position (61%) and a preference for Val (44%) in the P4 position. In the P2 position, gzm A was more tolerating and accepted aromatic amino acids, as well as Gly, Leu, and even negatively charged amino acids (**Figure 4**). The results from the phage display analysis of the target selectivity for gzm A and K is also visualized in the form of Web-logo figures (**Figures 4C, D**).

### 3.4 Verifying the consensus sequence by the use of recombinant protein substrates

**Figure 5:**
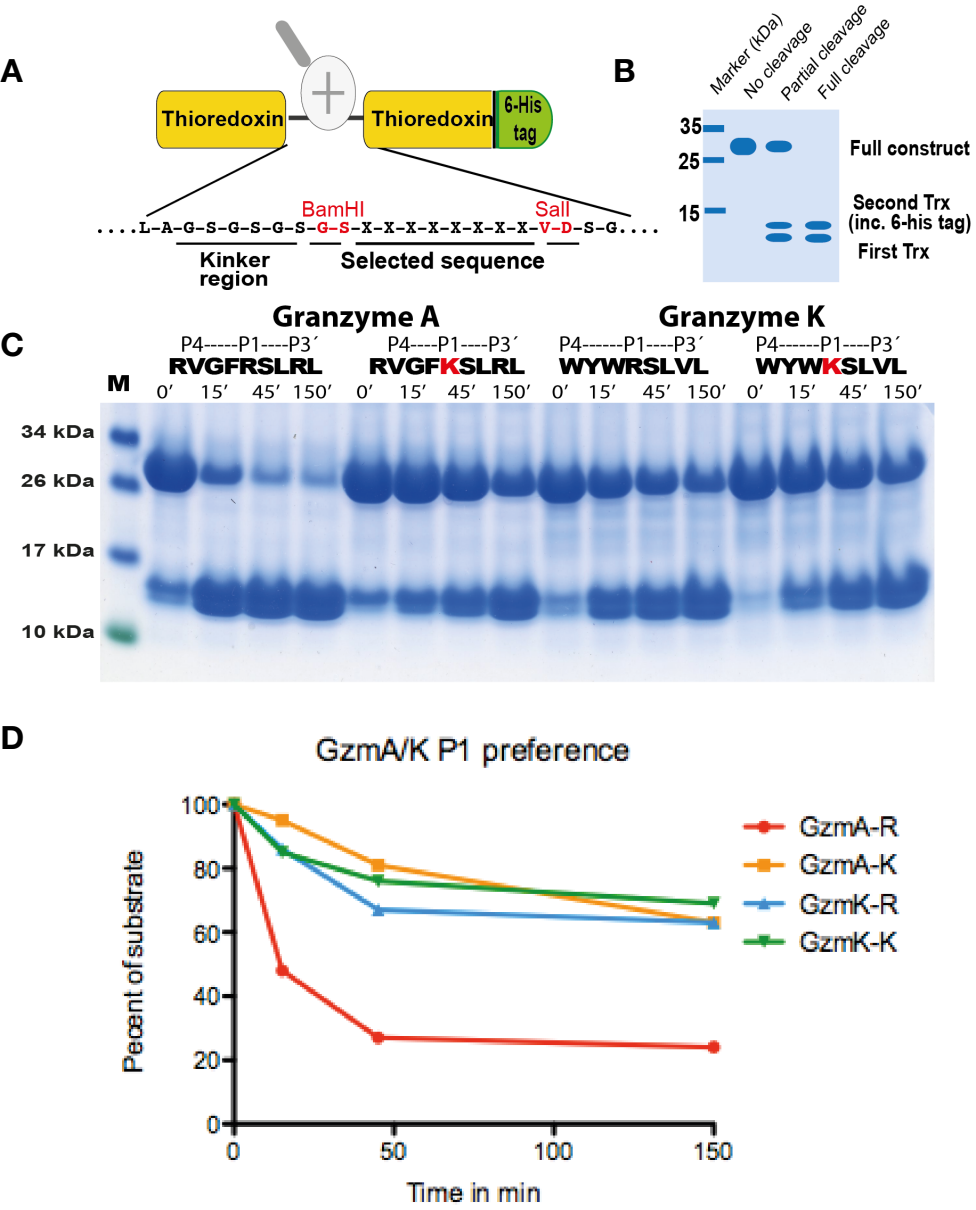
Analysis of the selectivity of human gzms A and K against substrates with an Arg or a Lys in the P1 position. In **(A)**, the overall structure of the recombinant protein substrates used for the analysis of the cleavage by gzm A is depicted. The sequences analyzed were positioned between two thioredoxin molecules with a His_6_-tag attached to the C terminal of the second Trx molecule. Two unique restriction sites (BamHI and SalI) were used for the insertion of the target sequences between the two thioredoxin molecules. The major steps of the analysis are shown in **(A)**. A schematic example of this type of analysis is depicted in **(B)** explaining various possibilities of the cleavage patterns. **(C)** shows the cleavage with Arg or Lys containing substrates with gzm A and gzm K The first two substrates with gzm A and the following two substrates with gzm K The substrates used for cleavage are the consensus substrates for the respective enzyme with an Arg in the P1 position and the second substrate are a substrate where the Arg has been exchanged for a Lys, marked in red. All protein gels were analyzed using the UN-SCAN-IT Gel Analysis Software from Silk Scientific Inc. (Orem, Utah USA). By scanning we measure the remaining amount of the 2xTrx protein in percent compared to the starting material after 15, 45 and 150 minutes of cleavage. The scanning result is shown in **(D)**. By the densitometric scanning and comparing number of minutes to get to the same percentage of remaining material we estimate a 15 times difference in cleavage efficiency between an Arg and a Lys in the P1 position for gzm A and a 3.75 times difference for gzm K.

**Figure 6:**
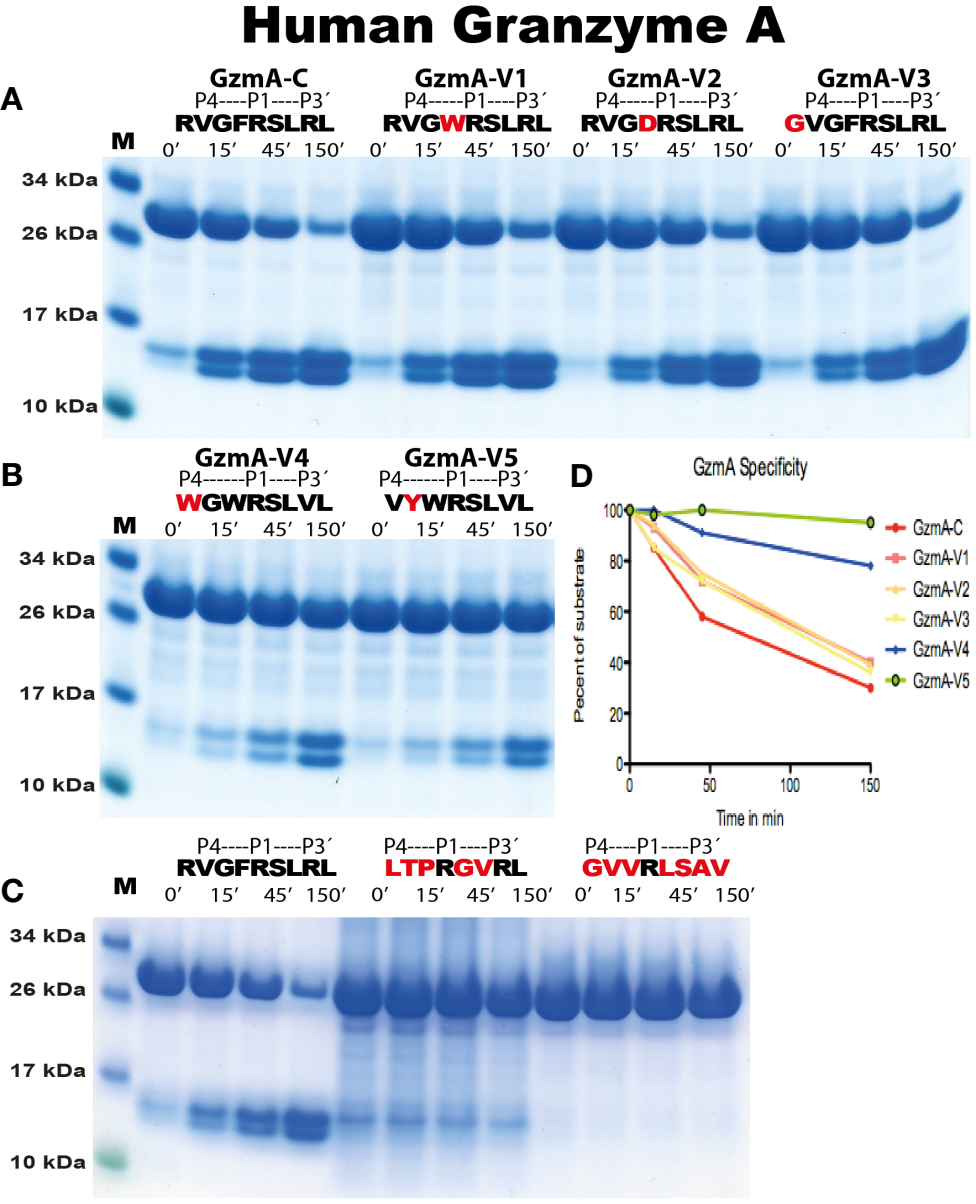
Analysis of the cleavage specificity of human gzm A by the use of recombinant protein substrates. The cleavage of substrates by human gzm A is shown in **(A-C)**. The sequences of the target region and cleavage time (minutes) were indicated above the lanes of different samples. The bands between 26kDa and 34kDa represent un-cleaved substrates and those between 10kDa and 17kDa represent cleaved substrates. The difference in size of cleaved bands was caused by the His_6_-tag of one of the Trx molecules. All protein gels were analyzed using the UN-SCAN-IT Gel Analysis Software from Silk Scientific Inc. (Orem, Utah USA). By the densitometric scanning, shown in **(D)**, and comparing number of minutes to get to the same percentage of cleavage we estimate a minor approximately 30% reduction in cleavage of variants V1, V2 and V3 compared to the consensus substrate (RVGFRSLRL), whereas variants V4 and V5 show a 10 and 50 times lower cleavage rate compared to consensus, respectively.

**Figure 7:**
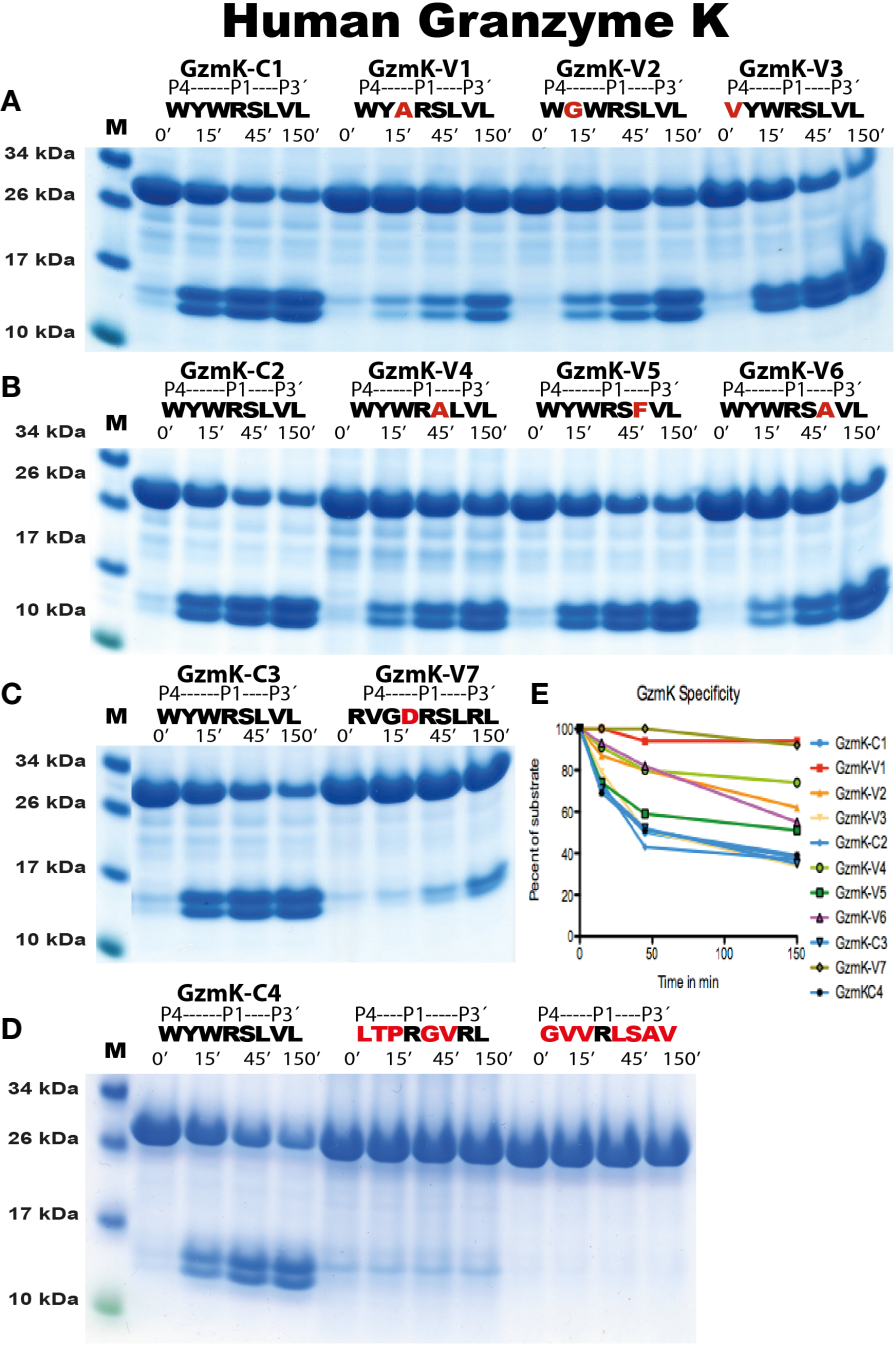
Analysis of the cleavage specificity of human gzm K by the use of recombinant protein substrates. The cleavage of substrates by human gzm K are shown in **(A-D)**. The sequences of the target regions and cleavage time (minutes) were indicated above the lanes of different samples. The bands between 26kDa and 34kDa represent un-cleaved substrates and those between 10kDa and 17kDa represent cleaved substrates. The difference in size of cleaved bands was caused by the His_6_-tag of one of the Trx molecules. All protein gels were analyzed using the UN-SCAN-IT Gel Analysis Software from Silk Scientific Inc. (Orem, Utah USA). By the densitometric scanning, shown in **(E)**, and comparing number of minutes to get to the same percentage of cleavage we estimate a major approximately 30 times lower cleavage rate for variant 1 (V1), a six times difference for V2, no difference for V3, a 10-15 fold difference for V4, a three-fold difference for V5, a 3-4 fold for V6 and a 50 fold difference for V7. The high reproducibility in the assay can also be seen from the four samples of the consensus sequence that was run at different times, C1, C2, C3 and C4, which all gave almost identical scanning results, all shown in blue.

To verify the sequences obtained from the phage display analysis, and to obtain quantitative information concerning the importance of particular amino acids at and around the cleavage site, recombinant protein substrates were used. The consensus sequence from the phage display analysis and several variants of these sequences were designed and produced as recombinant substrates in a two-thioredoxin (2xTrx) system (**Figure 5A**). Double-stranded oligonucleotides encoding the consensus substrates and a number of variants of these substrates were designed, ordered, and ligated into the 2xTrx substrate vector. A His_6_-tag was added in the C-terminal of the second Trx molecule for easier purification (**Figure 5A**). The vectors carrying the target sequences were transferred into *E. coli Rosetta gami* for expression and purification. These purified 2xTrx proteins were used to analyze the specificity of human gzm A and K (**Figures 5**-**7**). All protein gels were quantified by gel scanning using the UN-SCAN-IT Gel Analysis Software and the times in minutes to obtain the same amount of cleavage was compared.

The analysis of the human gzms A and K with the recombinant substrates confirmed the preference for Arg over Lys in the P1 position as observed from the phage display analysis (**Figure 5**). Arg is preferred over Lys by a factor of 15 for gzm A and by a factor of 3-5 by gzm K (**Figure 5**). We could also confirm the marked difference in preference for amino acids N-terminally of the cleavage site (**Figures 6**, **7**). Gzm A did not efficiently cleave substrates with large aromatic amino acids in the P3 and P4 positions but accepted both aromatic and negatively charged amino acids in the P2 position (**Figures 6A, B**). There was actually a very strong effect of inserting aromatic amino acids in the P3 and P4 positions. We observed a 10 to 50-fold lower cleavage rate of when a Trp was inserted in the P4 position and a Tyr in the P3 position respectively (**Figure 6**). In contrast, gzm K showed very weak activity towards substrates containing negatively charged amino acids in the P2 position (**Figure 7C**). Insertion of an Asp in the P2 position resulted in a dramatic drop in cleavage, estimated to an almost 50-fold reduction (**Figure 7C**). Insertion of the small amino acid Ala in the P2 position also resulted in an approximately 30-fold reduction in cleavage activity compared to the optimal Trp (**Figure 7A**). A Gly in the P3 position resulted in a reduction in activity by a factor of 6, but an insertion of Val in the P4 position was not negatively affecting the cleavage (**Figure 7A**). Replacing the Ser in the P1´position with an Ala resulted in a 10-15 fold decrease in cleavage rate but, unexpectedly, replacing the strongly favored Leu, from the phage display analysis, in the P2´position with a Phe or an Ala resulted in only a 3 fold drop in cleavage activity (**Figure 6**).

We also tested two substrates that we have produced for other enzymes with primary specificity for Arg, one of them is the consensus cleavage site for the coagulation enzyme thrombin (**Figures 6**, **7**) (28). No cleavage was seen for any of these substrates for either gzm A or K, showing the relatively high specificity in target selection for both enzymes (**Figures 6C**, **7D**).

### 3.5 Analysis of the cytokine inducing activity of recombinant active human gzm A on monocytes

**Figure 8:**
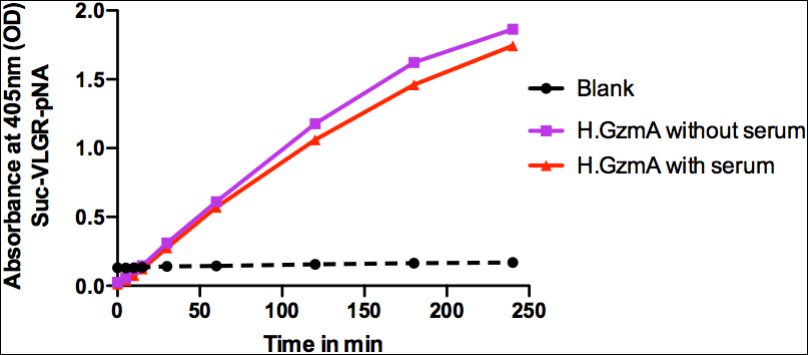
Granzyme A activity in the presence and absence of 5% fetal calf serum. The activity of the recombinant human gzm A was analyzed in cell culture medium with or without 5% fetal calf serum using the chromogenic substrate (Suc-VLGR-pNA).

Human monocytes were purified from buffy coats using magnetic beads coated with a monoclonal antibody against CD14 to study the potential cytokine inducing activity of human recombinant gzm A. Active recombinant human gzm A was added to *in vitro* cultures of these purified monocytes and the expression of the classical inflammatory cytokines IL-1α, IL-1β, IL-6 and TNF-α, was studied by analysis of their transcriptome. Gzm A was found to induce expression of all the classical inflammatory cytokines listed above. However, the cytokine-inducing activity of 10 µg of enzymatically active gzm A was only a few percent of the corresponding activity of 1µg of *E. coli* LPS (**Table 1**). Moreover, the induction of the same cytokines as LPS made us suspect that the inducing activity could actually come from a small amount contaminating LPS in the recombinant gzm A. We therefore analyzed the LPS content in the granzyme A preparation and found that the recombinant enzyme that we added to the culture contained 0.1 ng of LPS, which indicate that at least parts of the cytokine induction in these monocytes could be due to the contaminating LPS. The activity of the recombinant human gzm A was also analyzed in the presence and absence of 5% fetal calf serum in cell culture medium, using the chromogenic substrate (Suc-VLGR-pNA), to ensure that enzyme was active during the culture conditions used in this study. The recombinant human gzm A was found to be highly active during the two and a half hours analyzed (**Figure 8**). This analysis also showed that the enzyme is only minimally affected by the plasma protease inhibitors found in fetal calf serum (**Figure 8**).

**Table 1:** Ampliseq analysis of the expression of five inflammatory cytokines and chemokines during *in vitro* culture for 4 hours of human monocytes in the presence or absence of 1 ug/ml of LPS (4h LPS) or 10 ug of recombinant active human gzm A (4h GzmA).

	0	4h	4h-LPS	4h Gzm A
IL-1α	0	6	2745	56
IL-1β	12	72	43189	638
TNF-α	86	61	2955	271
IL-6	0	1	8386	20
IL-8	176	181	46829	2418

Controls included cell cultured for 4h in culture medium without any additions (4h) and cells analyzed before *in vitro* culturing (0), directly after purification according to previously published procedure (26).

### 3.6 Transcriptome analysis of the expression of granzymes in four different human peripheral blood cell populations

**Figure 9:**
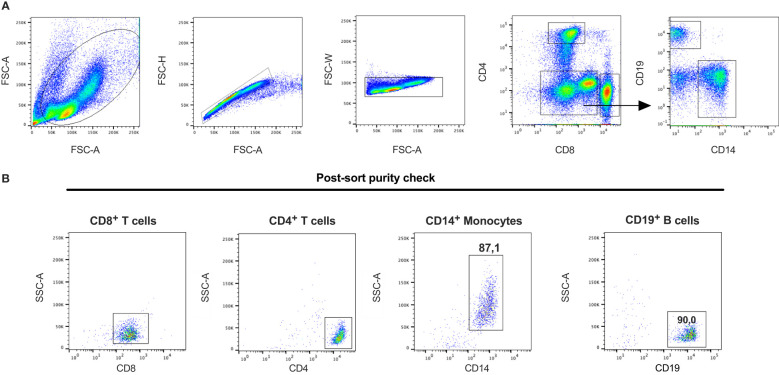
Gating strategy and post-sort purity check for the isolation of human peripheral blood cell subpopulations. Blood samples collected from buffy coats were used to purify peripheral blood mononuclear cell populations. Cells were stained with antibodies against CD4, CD8, CD14 and CD19 to isolate CD8^+^ T cells, CD4^+^ T cells, CD19^+^ B cells and CD14^+^ monocytes. **(A)** Gating strategy to identify the different subpopulations. **(B)** A fraction of sorted cells were re-analyzed by flow cytometry after the sort to determine the purity of each isolated cell population. The figure is a representative sample of the two different samples used for the experiments.

To study the level of expression of the human gzms and in particular gzm A and K in CD8^+^ T cells compared to other human peripheral blood immune cell populations, human CD19^+^ B-cells, CD14^+^ monocytes and both CD4^+^ and CD8^+^ T cells were isolated from PBMCs from two human blood donors (**Figure 9**). A quantitative analysis of their transcriptome was performed by the use of the Ampliseq technology. The CD8^+^ T cells was found to express low levels of transcripts for all the five different human gzms, and for perforin (PRF1) and granulysin (GNLY) (**Table 2**). Also the CD4^+^ T cells was found to express gzms A, K and M, and of PRF1 and GNLY, but not gzms B and H (**Table 2**). However, the expression levels in the CD4^+^ T cells were 2-5 times lower than in the CD8^+^ cells. The perforin levels were also very low in the CD4^+^ population indicating a lower cytotoxic activity (**Table 2**). Although, the levels in CD8^+^ T cells were higher than in the CD4^+^ cells they were very low compared to what we previously have observed for mast cells (29). It is at least 30-50 times lower levels in these T cells compared to mast cells indicating that the majority of the granzymes is being expressed upon T cell activation and that we only see a low basal level in circulating resting T cells. We also looked at the expression levels of a number of cell surface molecules and a transcription factor for T cells and other human immune cells to look closer at the potential heterogeneity in the isolated CD4^+^ and CD8^+^ T cell populations (**Table 2**). The results from this latter analysis is discussed in detail in the discussion section.

**Table 2:** Ampliseq analysis of four different peripheral blood immune cell populations.

Gene	B-cell	B-cell	Mono- cyte	Mono- cyte	CD4 T-cell	CD4 T-cell	CD8 T-cell	CD8 T-cell
Granule Proteases								
**GZMA**	0.1	0	0.4	0.1	**68**	**38**	**219**	**268**
**GZMK**	0	0	0	0.1	**148**	**51**	**228**	**146**
**GZMH**	0	0	0	0	9	0.4	280	103
**GZMB**	0.4	0.1	0	0	2	0.6	155	70
**GZMM**	0	0	0	0.2	**65**	**41**	**160**	**279**
**PRF1 (perforin)**	0.1	0	0	0	**41**	**15**	**274**	**632**
**GNLY (Granulycin)**	0.7	0.8	6	2	**109**	**50**	**722**	**205**
**FOXP3**	0	0.3	0	0.1	**30**	**23**	2	0.2
**GATA3**	0.1	0.4	0.4	0	**196**	**174**	**150**	**281**
**TBX21 (T-bet)**	5	3	0	0	**27**	**15**	**78**	**21**
**CCR7**	**83**	**52**	0	0	**124**	**129**	**145**	**286**
**CD44**	4	2	4	4	3	4	2	14
**SPN (CD43)**	0	0	0.9	0.7	**20**	**10**	**52**	**29**
**KLRG1**	3	4	0.4	0.9	**101**	**97**	**197**	**178**
**NKG2D(KLRK1)**	**34**	**19**	0.3	0	5	0.4	641	424
**NCAM1 (CD56)**	0	0.2	3	6	0.7	2	**17**	**21**
**KLRB1 (NK1.1)**	0.2	0.3	0.5	0	**504**	**245**	**420**	**183**

CD19^+^ B-cells, CD14^+^ monocytes and both CD4^+^ and CD8^+^ T cells were analyzed for their expression of a panel of T cell granule proteins and cell surface markers and transcription factors of T cells and other immune cells including the granzymes, perforin, granulycin, FOXP3, GATA3, T-bet (TBX21), CCR7, CD44, CD43, KLRG1, KLRK1, NCAM1(CD56) and KLRB1 (NK1.1). The number of reads, which directly correspond to their expression level, is presented for two individual samples originating from two different blood donors for each cell population. Among these four cell populations only the T cells express these granule proteins. Compared to lysozyme expression in monocytes (approximately 20 000 reads), the expression level of granzymes, perforin and granulysin, in T cells are relatively low. The bold is for values of 10 or higher to highlight the highly expressing cells.

## 4 Discussion

Analysis of the primary and extended specificity of both human gzms A and K shows that both of these enzymes prefer Arg over Lys, by a factor of approximately 15 in the P1 position for gzm A and by a factor 3-5 for gzm K, thereby being relatively strict Arg specific trypsin-like enzymes. One hundred percent of the sequences in the phage display analysis contained Arg in the P1 position, for both enzymes, and not a single sequence had a Lys in this position (**Figure 4**). The major differences between the two enzymes are instead located in their preference for particular amino acids just N-terminally of the cleavage site. Gzm A prefers small aliphatic amino acids such as Gly primarily in the P3 position, whereas gzm K prefers large aromatic amino acids in several positions N-terminally of the cleavage site. We could observe a high frequency of Trp, Phe, and Tyr in the P2, P3, and P4 positions for gzm K whereas Gly and Val were preferred amino acids in the P3 and P4 positions for gzm A. Gzm A showed a relatively relaxed selectivity in the P2 position where gzm K showed its strongest preference for aromatic amino acids (**Figures 4**-**7**). Gzm A also accepts negatively charged amino acids in the P2 position, whereas an Asp in this position results in an almost 50-fold reduction in cleavage activity for gzm K (**Figures 6**, **7**).

A detailed analysis of the specificity of gzm A has previously been performed using both cleavage of whole cell extracts, MS-analysis and by analysis of the cleavage of peptides (22). Another study used both total cellular extract and peptides on a chip (PepChip) arrays involved a comparative analysis of the specificities of human gzm A and K (21). Both of these studies also showed a clear preference for Arg over Lys in the P1 position of substrates (21, 22). However, in these analyses, a number of Lys substrates were also observed and for the extended specificity, a clear distinction compared to our studies. These previously published studies identified a number of gzm A substrates that have Pro in the P2 position (21, 22). In contrast, only very few P2 prolines were found in our analysis (**Figure 4**). One potential explanation is the difference in substrates used in the analysis of whole-cell extracts (22). The phage display analysis is based on linear sequences, where the best, most effectively cleaved, substrates are strongly favored. In contrast, in whole-cell extract, which consists of properly folded proteins, the cleavage is also highly dependent on the accessibility of the cleavage site. We have previously shown that accessibility is very important for one of the major mast cell enzymes, the human chymase, and also for the different pancreatic digestive enzymes, trypsin, chymotrypsin, and pancreatic elastase (30, 31). The amino acid Pro induces a bend in the structure and could therefore be overrepresented on the surface of a target protein. By being present on the surface the site would also be more accessible. This is most likely the major explanation for the difference between our study and previous studies (21, 22). One of our substrates in the 2xTrx assay actually has a Pro in the P2 position and represents the consensus substrate for human thrombin (LTPRGVRL) (**Figure 6C**) (27). This substrate was a very poor substrate for human gzm A, and we could not detect any cleavage at all of this substrate, indicating that sequences with Pro in the P2 position are not favored substrates (**Figure 6C**). The same is true for human gzm K, where no cleavage was observed with the thrombin consensus substrate (**Figure 7D**).

There are also a few additional minor differences between our study and the studies by Bovenschen et al. and Van Damme et al. in preference for amino acids in positions P1´, P2´and P3 (21, 22). While Ser dominate in P1´, Leu in P2´ and Gly in P3 in our study the previous study suggests Ala, Gly and Ala in respective positions (22). In P3´we detect Leu while Gly is the major aa in the study by Van Damme et al. (22). Finally, in the P3 position, we see Gly while Van Damme et al. see Ala (22). However, in all three cases, the amino acid Van Damme et al. detect most frequently in their analysis is the second most preferred amino acid found in our study, indicating that the difference between our studies is minor (22). In the analysis of the sequences from the native proteins, they also observe an overrepresentation of sequences with a high percentage of charged amino acids, primarily negatively charged, at a distance C-terminally from the actual cleavage site. This difference could indicate an exosite interaction (22). Such negatively charged regions have an important role as exosite interacting regions in coagulation factors V and VIII for activation cleavage by thrombin (32). However, as the authors comment, due to lack of conservation in other regions of gzm A the likelihood of a conserved exosite is low, why this overrepresentation most likely is due to surface location where charged residues are common (22).

Recently two articles on the development of fluorogenic substrates and specific inhibitors of human gzms A and B have resulted in interesting data concerning the cleavage preference for these two enzymes (33, 34). The analysis of substrate preference for gzm A show in full agreement with our phage display result a strong preference for Gly in the P3 position and a more relaxed selectivity in the P2 position of substrates (33). These newly developed substrates also make it possible to look closer at the *in vivo* function of these two important enzymes.

From our analysis, we conclude that the major difference in cleavage specificity between human gzms A and K primarily is found in residues located N-terminally of the cleavage site. These positions clearly indicate the selection for specific and not shared *in vivo* substrates for these two enzymes. The clear separation of the two proteins in the phylogenetic tree also strengthens such a conclusion. However, we cannot yet say what that difference in substrates means for the *in vivo* function, a question of high interest for their role in T cell biology.

The accumulated information concerning the role of gzm A in the induction of inflammation is still connected with several inconsistencies, as described in the introduction. There are both experimental data published that shows that proteolytic activity is needed for the induction of cytokine and chemokine expression and other published information that say that this induction is independent of proteolytic activity (7, 14).

Based on the results from one of our recent studies on human monocytes and their response to small amounts of bacterial lipopolysaccharides (LPS), we find a high risk that the cytokine/chemokine induction is not, or only partly, dependent on gzm A (26). An alternative explanation is that this induction instead primarily is dependent on contaminating LPS in the protease preparations. Our previous study shows that human peripheral blood monocytes are extremely sensitive to LPS, and produce massive amounts of transcript for all the classical inflammatory cytokines and chemokines, such as IL-1, IL-6, and TNF-α, and also of the chemokine IL-8, after only four hours of cultivation in the presence of LPS (26). Exactly the same cytokines and chemokines were reported as upregulated when adding gzm A to the monocytes (7, 14). Due to the fact that LPS very easily can contaminate pipettes and solutions, and that monocytes are extremely sensitive to even low amounts of LPS, makes us question the existing literature on the role of gzm A in the induction of inflammatory cytokines and chemokines. We have also performed an analysis of the effect on the monocyte transcriptome by the addition of 10 ug of active purified human gzm A to the cell medium, and after 4 hours of incubation only detected a minor increase in the expression of the inflammatory cytokines and chemokines compared to adding LPS (**Table 1**). Addition of 1 ug/ml of LPS resulted in an increase in IL-6 mRNA levels by more than 75 000 times after only 4 hours of culturing (**Table 1**) (26). IL-8 became the dominating transcript, exceeding lysozyme by 50%, which was the most abundant transcript before LPS stimulation (26). It is therefore likely that the minor increase in these cytokines and chemokines after addition of gzm A in our study is due to a minor LPS contamination. We have used recirculating glass pipettes during culturing of the cell lines for the production of the recombinant granzyme A. A small amount of LPS may remain after eluting the gzm A from the Ni^2+^ IMAC columns used during purification. We have tested the amount of LPS in the preparation with the endotoxin test and seen that 10 ug of gzm A added to the culture correspond to approximately 0.1 ng of LPS. The question of the role of granzyme A in the induction of inflammatory cytokines and chemokines is therefore still not fully resolved. However, what we can conclude is that if granzyme A has a role in cytokine and chemokine induction, it is then relatively minor compared to LPS (**Table 1**). We can also confirm that the granzyme A is highly active during the condition used in the study, including the presence of 5% fetal calf serum as shown in **Figure 8**. This result also indicates that the inactivation of granzyme A by plasma protease inhibitors is a relatively slow process.

Transcriptome analysis of freshly isolated blood cells also showed expression of all five gzms in CD8^+^ T cells and also expression of three of them in CD4^+^ cell, the gzms A, K and M (**Table 2**). However, the expression in the CD4^+^ cells were considerably lower than in the CD8^+^ cells, only 20-50% of the levels in CD8^+^ cells (**Table 2**).

All the four peripheral blood cell populations of this study represent a mix of different subpopulations of cells. The B cells are a mix of B1 and B2 B cells, the monocytes of CD16^+^ and CD16^-^ cells and the T cells of circulating resting cells, memory cells, regulatory cells, effector T cells and possibly NKT cells. For the B cells and monocytes this is of lower importance for this study as both of these cell populations do not express granzymes or perforin (**Table 2**). However, the heterogeneity of both the CD4^+^ and the CD8^+^ T cells has a clear impact of the analysis. Earlier single cell studies by four color flow cytometry (FACS) of the expression of gzms A, K and B in CD4^+^ and CD8^+^ T cells have shown the presence of gzm A in 74%, of gzm B in 51% and of gzm K in 24% of the CD8+ T cells (35). Almost all the gzm K positive cells also expressed gzm A whereas only very few (4%) of the gzm K positive cells expressed gzm B (35). Almost 100% of the gzm B expressing cells also expressed gzm A indicating that almost all gzm expressing cells expressed gzm A (35). Interestingly, the gzm K expressing CD8^+^ T cells were almost all (88%) positive for CD28. In contrast to only 20% of the gzm B positive cells that may tell something about their state of activation (35). Almost 40% of the gzm K positive cells also seems to lack expression of perforin, which indicate non-apoptotic function of these cells (35). Within the γδ-T cells almost all cells, over 90%, were gzm positive (35). In contrast, very few of the CD4^+^ T cells expressed gzms (35). Only 3.3% were positive for gzm A, 0.9% for gzm B and 2.4% for gzm K (35).

Another FACS analysis of human peripheral blood mononuclear cells showed similar results concerning the expression of gzms in CD4^+^ and CD8^+^ T cells (36). Approximately 52% of the CD8^+^ T cells were gzm A positive and 37% gzm B positive indicating that a majority of CD8^+^ cells are gzm positive, whereas only 3.4% of the CD4+ T cells were positive for gzm A and only 1.2% for gzm B (36). Interestingly, anti CD3 and anti CD46 stimulation resulted in an upregulation of gzm B but not A in almost all cell in the CD4^+^ T cells (36). Somewhat contradicting information concerning the expression of gzm B is also coming from another study of human immune cell types where CD4^+^ memory cells express gzm B, which is in contrast to our analysis where we see no or very very low levels of gzm B in the CD4^+^ T cells (**Table 2**) (37).

The natural question is then what minor subpopulation of CD4^+^ T cells is it that express gzms? One possibility is that CD4 positive NKT cells is the source of the granzymes as they are functionally more similar to CD8^+^ T cells. However, NKT cells are considered a very minor population of cells, less than 1% of the circulating blood T cells and does thereby only minimally contribute to the mRNA levels in these peripheral blood T cell populations (38). In our analysis the CD4^+^ T cells also expressed very low levels of perforin compared to the CD8^+^ cells indicating that they have lower potency as apoptosis inducing cells (**Table 2**). Cytotoxic CD4^+^ T cells have also been described, and that these cells can significantly increase in numbers in elderly people compared to young adults (39). They are characterized by expression of perforin, gzm B and NKG2B (KLRK1) (39). Neither granzyme B nor NKG2B were expressed at significant levels indicating that cytotoxic CD4^+^ T cells are essentially absent in our two samples of cells (**Table 2**).

To look closer at the heterogeneity of these T cells we include the expression of a central marker for regulatory T cells, the FOXP3 transcription factor, and the dominating transcription factors for TH2 and TH1 type of T helper cells GATA3 and T-bet, respectively (**Table 2**). Only low level of expression of FOXP3 was seen in the CD4^+^ T cells indicating that only a minor fraction of these circulating T cells are regulatory T cells (**Table 2**). Low levels of T-bet was also observed compared to GATA3 indicating as expected that TH2 type dominate over TH1 cells (**Table 2**) Central memory T cells have been shown to express the chemokine receptor CCR7 and CD44 (40). We can here show that CCR7 is expressed at relatively high levels in both the CD4^+^ and the CD8^+^ T cells (**Table 2**). CCR7 has been shown to be expressed also on naïve T cells questioning CCR7 as a good marker for only memory cells (40). In contrast CD44 is expressed at very low levels, and we detect it only in one of the CD8^+^ cell samples indicating low levels of central memory cells in both the circulating CD4^+^ and CD8^+^ T cells (**Table 2**). Most memory cells also seem to be located in lymphoid and non-lymphoid tissues and not in the circulation (40).

To look for the presence of effector T cells which have been shown to express perforin gzm B, CD43 and KLRG1 we looked at the expression levels of CD43 and KLRG1 in these cells (40). By looking at the expression of CD43 (SPN) and KLRG1 we can see a low level of expression of CD43 and higher levels of KLRG1 in both CD4^+^ and CD8^+^ cells indicating that we have a fraction of T effector cells in the circulation and that they may be the source of both the granzymes and perforin.

Several studies have also shown the presence of granzymes A, K and B in different NK cell populations (35, 36). Most circulating CD56^+^ and NK1.1^+^ NK cells were in one of these studies shown to express both gzms A and B (36). We can here show that the NK cell marker NK1.1 (KLRB1) is found at relatively highly levels in both the CD4^+^ and the CD8^+^ cells (**Table 2**). However, the CD56 was found at only low levels in the CD8^+^ T cell sample possibly indicating a low number of CD8^+^ NKT cells in this sample (**Table 2**) (36). The levels of the granzymes seems also to vary between individuals making the picture even more complex (35).

The functional consequences of the complex pattern of expression of both gzms A and K in both CD4^+^ and CD8 ^+^ cells is difficult to explain but may give additional clues to the role of these two granzymes in T cell biology. The fact that a large fraction of the gzm K positive CD8^+^ T cells lacked perforin, and that CD4^+^ cells in general play a very minor role in the induction of apoptosis in infected cells points to a minor role of gzms A and K in apoptosis induction. Compared to LPS they are also very weak inducers of cytokines indicating a totally different role for gzm A, which is neither apoptosis nor potent cytokine inducer.

Major questions apparently remain concerning the biological functions of gzms A and K. If not being a potent inducer of inflammatory cytokines and chemokines what is then the primary role of gzm A? To this comes the question of what difference exists in targets for mammalian gzms A and K? By using the consensus cleavage sequences for gzms A and K for comparison with the entire human proteome, the increased detail in the extended cleavage specificity of both human gzms A and K can now provide important tools in screenings for the primary targets for these two enzymes. Such future studies may, after a detailed cleavage analysis of potential novel targets identified through this screening, reveal the biological function of gzms A and K and shed new light on the evolutionary conserved role of these enzymes in the immune defense.

## Data availability statement

The original contributions presented in the study are included in the article. Further inquiries can be directed to the corresponding author.

## Ethics statement

Ethical review and approval was not required for the study on human participants in accordance with the local legislation and institutional requirements. Written informed consent for participation was not required for this study in accordance with the national legislation and the institutional requirements. Ethical approval was not required for the studies of human cells because these cells came from unidentifiable blood donors.

## Author contributions

Involved in the conception or design of the work: EA, JR, LH. Data acquisition: EA, JR, EE. Analysis of the data: EA, JR, ZF, SA, EE, LH. Writing and reviewing: EA, JR, ZF, SA, EE, JH, SW, A-KO, LH. All contributing authors critically revised the article, contributed to the article, and approved the submitted version.
